# Seventeen-Armed Star Polystyrenes in Various Molecular Weights: Structural Details and Chain Characteristics

**DOI:** 10.3390/polym12091894

**Published:** 2020-08-23

**Authors:** Jia Chyi Wong, Li Xiang, Kuan Hoon Ngoi, Chin Hua Chia, Kyeong Sik Jin, Akira Hirao, Moonhor Ree

**Affiliations:** 1 Materials Science Program, Department of Applied Physics, Faculty of Science and Technology, University Kebangsaan Malaysia, Bangi 43600, Selangor, Malaysia; wongjiachyi@gmail.com (J.C.W.); ngoikuanhoon@gmail.com (K.H.N.); 2 Department of Chemistry and Pohang Accelerator Laboratory, Pohang University of Science and Technology, Pohang 37673, Korea; lea1990@postech.ac.kr; 3 Pohang Accelerator Laboratory, Pohang University of Science & Technology, Pohang 37673, Korea; 4 Department of Chemical Science and Engineering, Graduate School of Materials and Chemical Engineering, Tokyo Institute of Technology, 2-12-1-S1-13, Ohokayama, Meguro-ku, Tokyo 152-8550, Japan; 5 Department of Chemical Engineering, National Taiwan University, No. 1, Sec. 4, Roosevelt Road, Taipei 10617, Taiwan

**Keywords:** 17-armed star polystyrene, synchrotron X-ray scattering analysis, dynamic light scattering analysis, molecular structure, molecular size, size distribution, radial density profile, chain characteristics

## Abstract

Star-shaped polymers are very attractive because of their potential application ability in various technological areas due to their unique molecular topology. Thus, information on the molecular structure and chain characteristics of star polymers is essential for gaining insights into their properties and finding better applications. In this study, we report molecular structure details and chain characteristics of 17-armed polystyrenes in various molecular weights: 17-Arm(2k)-PS, 17-Arm(6k)-PS, 17-Arm(10k)-PS, and 17-Arm(20k)-PS. Quantitative X-ray scattering analysis using synchrotron radiation sources was conducted for this series of star polymers in two different solvents (cyclohexane and tetrahydrofuran), providing a comprehensive set of three-dimensional structure parameters, including radial density profiles and chain characteristics. Some of the structural parameters were crosschecked by qualitative scattering analysis and dynamic light scattering. They all were found to have ellipsoidal shapes consisting of a core and a fuzzy shell; such ellipse nature is originated from the dendritic core. In particular, the fraction of the fuzzy shell part enabling to store desired chemicals or agents was confirmed to be exceptionally high in cyclohexane, ranging from 74 to 81%; higher-molecular-weight star polymer gives a larger fraction of the fuzzy shell. The largest fraction (81%) of the fuzzy shell was significantly reduced to 52% in tetrahydrofuran; in contrast, the lowest fraction (19%) of core was increased to 48%. These selective shell contraction and core expansion can be useful as a key mechanism in various applications. Overall, the 17-armed polystyrenes of this study are suitable for applications in various technological fields including smart deliveries of drugs, genes, biomedical imaging agents, and other desired chemicals.

## 1. Introduction

Star-shaped polymers are structurally intriguing materials that consist of several linear polymer chains connected at one point (i.e., a central core). In fact, star-shaped polyamides with four and eight arms were reported in 1948 as the first star polymers, which were prepared by step-growth polymerization [1]. Star polystyrenes having three and four arms were introduced in 1962 as the first well-defined star polymers, which were synthesized by anionic polymerization [2]. Since these discoveries, star polymers have become attractive to both academia and industry because of the unique molecular topologies and unusual physical and chemical properties expected. As a result of much research effort that was carried out particularly during the last three decades, various synthetic schemes have been developed to provide well-defined star polymers; they were reviewed in several articles [3,4,5,6,7,8].

Star polymers were found to exhibit very interesting properties, such as smaller hydrodynamic radii, smaller radii of gyration, and lower viscosities, compared to the linear counterparts [4,5,6,7,9,10,11,12]. Crystalline star polymers revealed lower melt temperatures, lower crystallization temperatures and lower degrees of crystallinities than the linear counterparts [7]. These unusual properties might originate basically from the compactness of molecular structures and the geometrical constrain of arm chains in such compact structure. The unique properties of star polymers were also reported to vary with the length and number of arms in addition to their chemical structure [7,11]. Furthermore, the structure and properties were further influenced by their central core structures [12,13]. These results give very limited information but are still important indications that the structure and properties of a star polymer are changed sensitively by all molecular parameters, including chemical structural nature, core structure, arm length (i.e., molecular weight), arm number, arm chain flexibility or rigidity, and polydispersity. Despite the compact molecular structures, star polymers can own peripheral end groups and additional internal groups, depending on their molecular designs [3,4,5,6,7,8]. Both arm end groups and internal groups could be active due to either their own chemical natures or functionalizations [3,4,5,6,7,8]. Such peripheral and internal functional groups could expand the applicability of star polymers in various areas including lubrications [3,14], thermoplastic elastomers [15,16], catalysis [3], micro- and nano-electronics [17,18,19], cosmetics [3], drug and gene deliveries [3,4], biomedicals [3,4,20], and nanostructural materials [3,4,21,22,23]. Therefore, deeper understanding in the molecular structure and properties are still challenged for better applications and smarter molecular design of star polymers.

**Figure 1:**
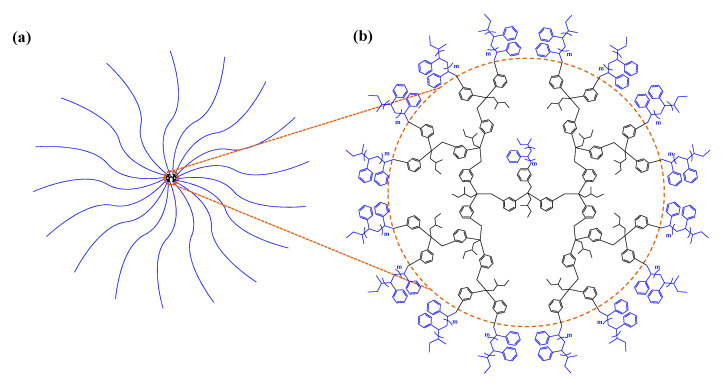
Chemical structure and characteristics of 17-armed star polystyrene: (**a**) schematic molecular structure representation; (**b**) chemical structure detail, which is composed of a dendritic core and 17 polystyrene arms.

In this study, we have made great efforts to obtain the molecular structure details and arm chain characteristics of a series of 17-armed star polystyrenes in various molecular weights which are essential for gaining insights into properties and finding better applications: 17-Arm(2k)-PS, 17-Arm(6k)-PS, 17-Arm(10k)-PS, and 17-Arm(20k)-PS (Figure 1; Table 1). The star polymers were examined in a theta (Θ) condition (cyclohexane (CHX) at 35.0 °C) as well as in a good solvent (tetrahydrofuran (THF) at 25.0 °C) by using synchrotron X-ray scattering and dynamic light scattering (DLS). The quantitative X-ray scattering analysis has been successfully performed, providing a comprehensive set of three-dimensional (3D) structural parameters and chain characteristics. Qualitative scattering analysis, as well as DLS analysis, gave additional information on the sizes. Overall, the star polymers all exhibit fuzzy ellipsoidal shapes. They reveal interesting 3D structural characteristics based on the individual arms’ unique behaviors. Excitingly, they demonstrate highly selective volume alterations (i.e., expansion and contraction) of core and fuzzy shell in the opposite direction through solvent changes, which can be a very powerful mechanism for storing and delivering (releasing) desired chemicals or agents. All results are discussed with considering molecular weight effects on geometrical molecular structure and arm characters.

## 2. Materials and Methods

A series of 17-armed star polystyrenes in various molecular weights were supplied from Prof. A. Hirao’s laboratory; each star polymer was synthesized by the coupling reactions of polystyrene anions (i.e., lithium 1,1-diphenylethylene-end-capped polystyrene (DPE-end-capped PS^−^Li^+^)) of a given molecular weight to polystyrene of a chosen molecular weight with one-end group in dendritic architecture which was multifunctionalized by bromobenzyl moieties (PS(BnBr)_16_) according to the method reported previously [8,24,25]: 17-Arm(2k)-PS, 17-Arm(6k)-PS, 17-Arm(10k)-PS, and 17-Arm(20k)-PS (Figure 1). The molecular characteristics of the dendritic-core-based star polystyrenes are summarized in Table 1. CHX (C_6_H_12_, >99%) was purchased from Sigma Aldrich Company (Seoul, Korea) and THF (C_4_H_8_O, 99.8%) was supplied by Samchun Chemical Company (Seoul, Korea).

For synchrotron X-ray scattering and DLS analyses, solutions (0.25−1.00 wt% concentration) of each star polymer were prepared in CHX and THF and then filtered by using disposable syringes equipped with polytetrafluroethylene filters (0.22 μm pore size). X-ray scattering measurements were carried out at the 4C beamline [26,27,28] of the PLS-II facility (a third-generation synchrotron radiation facility being operated at 3.0 GeV and 400 mA by Pohang Accelerator Laboratory, Pohang, Korea) by using an X-ray beam with a wavelength λ of 0.07336 nm and a two-dimensional (2D) charged-coupled detector (CCD: model Rayonix 2D SX 165, Evanston, IL, USA); the sample-to-detector distances (SDD) was 1.0 and 4.0 m. Quartz capillary cells with 1.5 mm outer diameter were used as solution sample cells; for each cell, around 70 μL solution sample was used. The polymer solutions in THF (good solvent) were exposed to the X-ray beam for 20 s at 25.0 °C (± 0.1 °C); the polymer solutions in CHX (Θ solvent) were exposed to the X-ray beam for 20 s at 35.0 °C (± 0.1 °C) (Θ condition). The scattering angles were calibrated by using a precalibrated Ti-SBA-15 (Sigma Aldrich Company, Seoul, Korea) and silver behenate (Tokyo Chemical Industry, TCI, Tokyo, Japan) as standards. The individual 2D scattering data were circularly averaged with respect to the X-ray beam center and then followed by normalizing to the transmitted X-ray beam intensity which was monitored with a scintillation counter positioned behind the sample. The scattering data was further corrected for the scattering arising from either THF at 25.0 °C or CHX at 35.0 °C. DLS measurements were performed at 25.0 °C (± 0.1 °C) for the polymer solutions in THF and at 35.0 °C (± 0.1 °C) for the solutions in CHX by using a Malvern DLS instrument (model: Zetasizer NanoZS90, Malvern Instruments Ltd., Worcestershire, UK) equipped with a He-Ne laser source of 632.8 nm wavelength and a detector at 90° scattering angle. Low-volume quartz batch cuvettes (model: ZEN2112, Malvern Instruments Ltd.) were used as sample cells.

## 3. Results and Discussion

**Figure 2:**
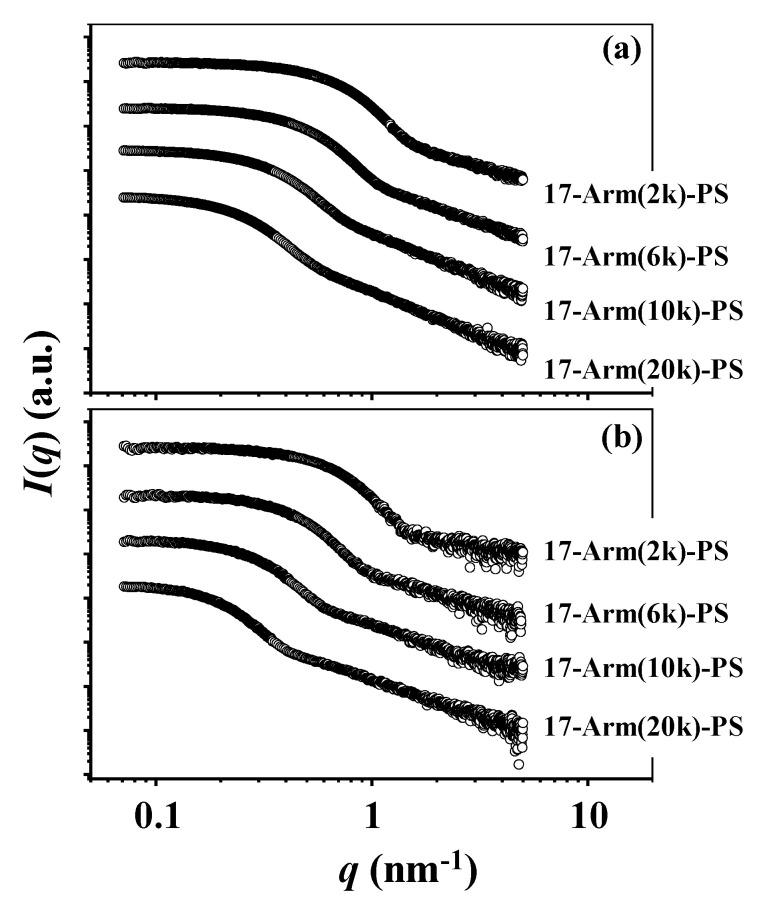
X-ray scattering data of 17-armed star polymers measured using an X-ray beam (λ = 0.07336 nm): (**a**) measured in Θ condition (cyclohexane (CHX) at 35.0 °C); (**b**) measured in a good solvent condition (tetrahydrofuran (THF) at 25.0 °C). *q* = (4*π*/λ)sin*θ* where 2*θ* is the scattering angle.

The 17-armed star polymers have been subjected to synchrotron solution X-ray scattering analysis in order to get insights into their 3D molecular structure and arm chain characteristics. Figure 2 displays representatives of the X-ray scattering data measured in CHX at 35.0 °C (Θ condition) and THF at 25.0 °C (a good solvent condition).

We have tried to analyze these scattering data first by using several conventional analysis schemes in qualitative manners below.

**Figure 3:**
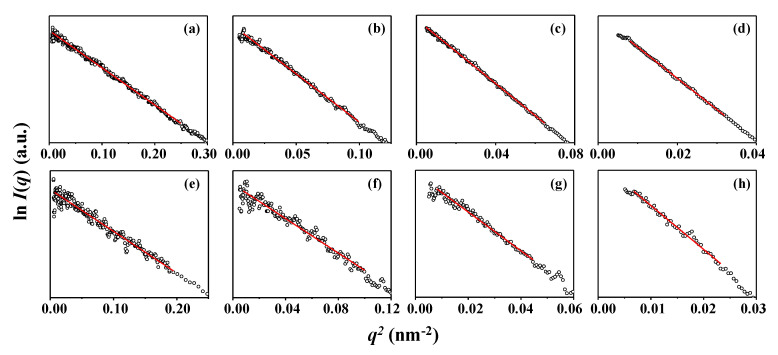
Guinier analysis results of the X-ray scattering data in Figure 2; CHX at 35.0 °C (Θ condition): (**a**) 17-Arm(2k)-PS; (**b**) 17-Arm(6k)-PS; (**c**) 17-Arm(10k)-PS; (**d**) 17-Arm(20k)-PS; THF at 25.0 °C (good solvent): (**e**) 17-Arm(2k)-PS; (**f**) 17-Arm(6k)-PS; (**g**) 17-Arm(10k)-PS; (**h**) 17-Arm(20k)-PS.

First, the scattering data has been analyzed by using the Guinier analysis scheme [29] valid under two boundary conditions, namely (i) spherical (or globular) scatterer and (ii) *q* regime (*q* = (4*π*/λ)sin*θ* where 2*θ* is the scattering angle) of less than 1.3/*R_g_* (here, *R_g_* is the radius of gyration of polymer), in order to get molecular size information on the star polymers; the detail of this analysis scheme is given in Supplementary Information. The scattering data in the low *q* regime are fitted well by the Guinier law, giving *R_g,G_* (Figure 3; Table 2). *R_g,G_* varies over the range 2.67−7.18 nm in the Θ condition and 2.80−8.52 nm in the good solvent, depending upon the molecular weights. Higher-molecular-weight 17-armed star polymer reveals larger size. All star polymers exhibit larger sizes in the good solvent than in the Θ solvent. The size expansion in the good solvent is 5% for 17-Arm(2k)-PS, 10% for 17-Arm(6k)-PS, 16% for 17-Arm(10k)-PS, and 19% for 17-Arm(20k)-PS. Namely, higher-molecular-weight 17-armed star polystyrene expands more in the good solvent.

**Figure 4:**
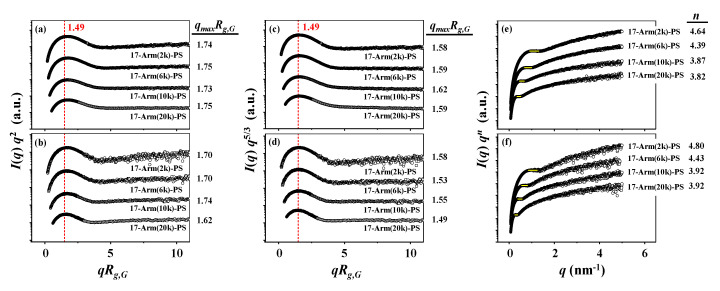
Kratky analysis results of the X-ray scattering data in Figure 2: (**a**,**c**,**e**) measured in CHX at 35.0 °C (Θ condition); (**b**,**d**,**f**) measured in THF at 25.0 °C (good solvent).

Second, the Kratky analysis scheme [30] has been applied in three different ways to the scattering data in the intermediate *q* regime in order to get information on the geometrical shape characteristics of the star polymers (details of the analysis scheme in Supplementary Information). The analysis results are shown in Figure 4 and Table 2. For 17-Arm(2k)-PS in the Θ condition, the overall shape of Kratky plot is quite different from that of a flexible linear polymer exhibiting Gaussian sphere as well as from that of a hard sphere (Figure 4a). In addition, a peak maximum appears at 1.74 (=*q*_max_*R_g,G_*), which is far from that (*q*_max_*R_g,G_* = 1.49) observed for isotropic sphere. Similar characteristics are observed in a modified Kratky plot (Figure 4c). The scattering intensity is found to follow a *q*^−4.64^ power law rather than *q*^−2^ and *q*^−5/3^ power laws (Figure 4e). Interestingly, similar scattering characteristics are discernible in the Kratky plots of the data measured in the good solvent (Figure 4b,d,f). Furthermore, similar scattering characteristics are also observed for the other star polystyrenes in the Θ condition as well as in the good solvent (Figure 4). These Kratky analysis results collectively inform that all 17-armed star polystyrenes in the Θ and good solvent conditions have very unique shapes, which are far from isotropic, Gaussian, and hard spheres. 

**Figure 5:**
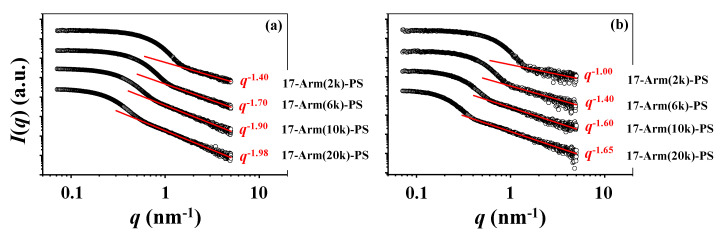
Porod analysis results of the X-ray scattering data in Figure 2 (**a**) CHX at 35.0 °C (Θ condition); (**b**) THF at 25.0 °C.

Third, the Porod analysis scheme [31] has also been considered for the scattering data in the high *q* regime in order to get information on the outmost surface of the star polymers (details of the analysis scheme in Supplementary Information). The star polymers in the Θ condition show scattering profiles obeying a *q*^−*n*^ power law with *n* = 1.40 to 1.98 in the high *q* region, depending on the molecular weights (Figure 5a). In the good solvent, they reveal scattering profiles following a *q*^−*n*^ power law with *n* = 1.00 to 1.65 in the high *q* region (Figure 5b). For these star polymers, the exponents are much smaller than 4 for the sharp and smooth surface of particle. These analysis results confirm that all 17-armed star polymers have much less sharp (i.e., much less dense) and smooth surfaces regardless of the Θ and good solvent conditions and the molecular weights. In addition, higher-molecular-weight star polymer has a higher degree of surface sharpness and smoothness.

**Figure 6:**
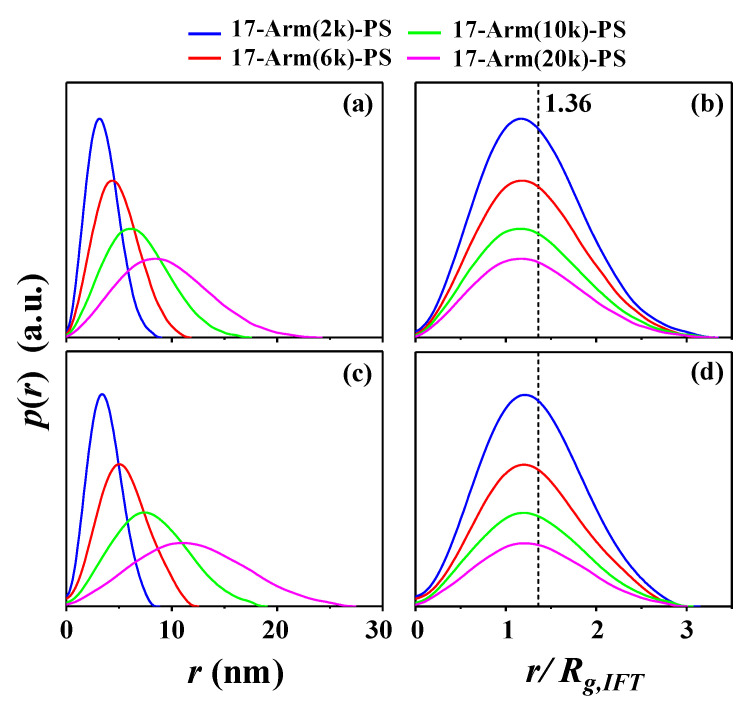
Indirect Fourier transformation (IFT) analysis results of the X-ray scattering data in Figure 2 (**a**,**b**) CHX at 35.0 °C (Θ condition); (**c**,**d**) THF at 25.0 °C (good solvent). *p*(*r*) is the pair distance distribution function.

Finally, the scattering data have been analyzed additionally by using the indirect Fourier transformation (IFT) method [32], which is a model independent analysis scheme; the detail of this method is given in Supplementary Information. Figure 6a,c illustrates the pair distance distribution function *p*(*r*) profiles obtained from the scattering data measured in the Θ condition and in the good solvent, respectively. Here, the pair distance distribution function describes the probability of finding two scatterers separated by a distance *r* inside a star polymer molecule. From each *p*(*r*) profile, the radius of gyration *R_g,IFT_*, radius at the peak maximum *R_max_*, and maximum size *D_max_* of star polymer have been extracted. The radial electron density distribution profile Δ*ρ*(*r*) has been additionally tried to be extracted. However, meaningful and realistic density profile could not be obtained, which might be caused from the assumption of monodispersity in size distribution in the IFT scheme. The obtained structural parameters are listed in Table 2. All *p*(*r*) profiles are asymmetric rather than symmetric in shape. The *R_max,IFT_/R_g,IFT_* values are much smaller than that (1.36) of an isotropic sphere (Figure 6b,d). Moreover, the *D_max,IFT_/R_max,IFT_* values are much larger than that (2.00) of a sphere. These results collectively confirm again that all 17-armed star polymers are geometrically far from spherical shapes in the Θ condition and more far from isotropic spheres in the good solvent. These analyses also confirm again that each star polymer has expanded volume in the good solvent when compared against that in the Θ condition: The size expansion in the good solvent against the Θ condition is 4% for 17-Arm(2k)-PS, 13% for 17-Arm(6k)-PS, 18% for 17-Arm(10k)-PS, and 26% for 17-Arm(20k)-PS.

**Figure 7:**
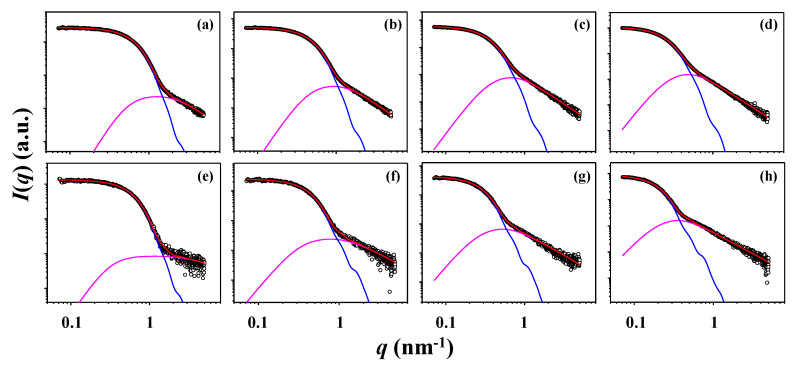
Quantitative fuzzy ellipsoidal model analysis results of X-ray scattering data in Figure 2 CHX at 35.0 °C (Θ condition): (**a**) 17-Arm(2k)-PS; (**b**) 17-Arm(6k)-PS; (**c**) 17-Arm(10k)-PS; (**d**) 17-Arm(20k)-PS; THF at 25.0 °C (good solvent): (**e**) 17-Arm(2k)-PS; (f) 17-Arm(6k)-PS; (**g**) 17-Arm(10k)-PS; (**h**) 17-Arm(20k)-PS. The open symbols are the measured data and the red solid line represents the sum of the profiles obtained by fitting the data using fuzzy ellipsoid model (blue line) and blob contributions (purple line).

**Figure 8:**
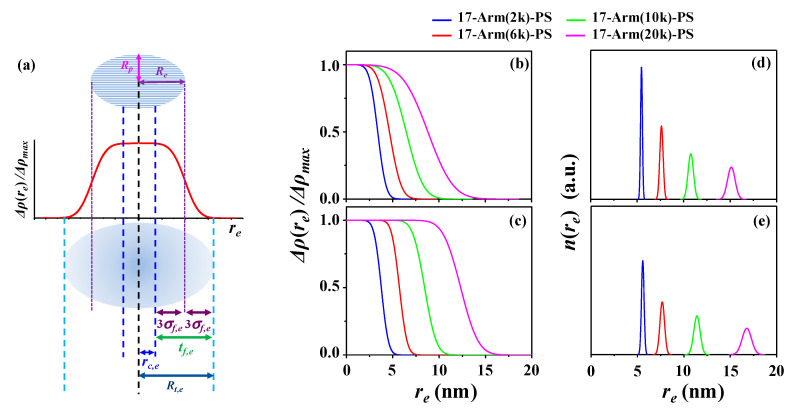
Fuzzy ellipsoidal structure, radial density profiles, and radial distributions determined by the quantitative analysis results of X-ray scattering data in Figure 7. (**a**) Oblate fuzzy ellipsoid in the front view of cross section and its structural parameters and radial density profile along the equatorial direction. Radial density profiles: (**b**) CHX at 35.0 °C (Θ condition); (**c**) THF at 25.0 °C (good solvent); radial distributions: (**d**) CHX at 35.0 °C (Θ condition); (**e**) THF at 25.0 °C (good solvent).

Taking into account the qualitative analysis results above, we have tried to further analyze the X-ray scattering data in a quantitative manner by using structural model approach, in order to get structural details on the star polystyrenes. In this quantitative analysis, we have considered several possible structural models and then found that the fuzzy ellipsoidal model is most suitable to analyze the scattering data successfully. As shown in Figure 7, all scattering profiles have been fitted satisfactorily with fuzzy ellipsoidal model together with the contribution of blobs (i.e., local density fluctuations) (see the structural model in Figure 8a); details of this model analysis is given in Supplementary Information. In addition, radial electron density distribution profiles Δ*ρ*(*r*) have been successfully extracted by the numerical Fourier transformation of the respective scattering amplitudes determined in this quantitative analysis (Figure 8b,c); the extraction detail of Δ*ρ*(*r*) is given in Supplementary Information. Moreover, the size distributions (namely, radial distributions) have been determined, as presented in Figure 8d,e. Pair distance distribution functions *p*(*r*) have also been extracted by numerical Fourier transformations of the extrapolated scattering intensity profiles obtained by the model analysis (Figure S1). All obtained structural parameters are listed in Table 2.

This quantitative analysis has found structural details of the star polymers below.

First, all 17-armed star polystyrenes exhibit fuzzy ellipsoidal shapes rather than spherical ones, regardless of the molecular weights as well as of the Θ and good solvent conditions. The mean equatorial radii *R_e_* are always much longer than the mean polar radii *R_p_*; here, *R_e_* and *R_p_* are defined under the assumption of isotropic ellipsoid (Figure 8a). For the Θ condition, the ellipsoidicity ratio *ε* (=*R_p_*/*R_e_*) is decreased a little bit from 0.51 to 0.46 by increasing molecular weight. In contrast, for the good solvent the *ε* value is increased slightly from 0.50 to 0.54 by increasing molecular weight. Overall, all star polymers have oblate ellipsoids in both the Θ condition and the good solvent. Considering the equivalent arm length of a 17-armed star polystyrene in Figure 1, the oblate ellipsoid shape might originate from the geometrical nature of the dendritic core part in the synthetic design.

Second, in the Θ condition, *R_e_* is 3.53 nm for 17-Arm(2k)-PS, 4.86 nm for 17-Arm(6k)-PS, 6.78 nm for 17-Arm(10k)-PS, and 9.30 nm for 17-Arm(20k)-PS. In the good solvent, *R_e_* is enlarged 9% for 17-Arm(2k)-PS, 19% for 17-Arm(6k)-PS, 26% for 17-Arm(10k)-PS, and 34% nm for 17-Arm(20k)-PS. These expansion trends in the good solvent are also confirmed by the averaged radii *R_av_* and radii of gyration *R_g_*.

Third, the pair distribution functions *p*(*r*) and their *R_max_* and *D_max_* values are almost identical to those obtained by the IFT analyses of the scattering data (Figure S1; Figure 6), confirming again that the quantitative model analyses of the scattering data were done successfully. In particular, the *R_g_* values are very close to those estimated by the Guinier and IFT analyses. These comparisons indicate that the Guinier and IFT schemes are reasonably good tools to determine the radii of gyration for the star polystyrenes of this study.

Fourth, a characteristic parameter *σ_f,e_* is determined along the equatorial direction for the fuzziness (i.e., fuzzy part); here, higher *σ_f,e_* indicates the presence of thicker fuzzy part in the ellipsoidal star polymer. In the Θ condition, *σ_f,e_* is 0.69 nm for 17-Arm(2k)-PS, 0.99 nm for 17-Arm(6k)-PS, 1.44 nm for 17-Arm(10k)-PS, and 2.11 nm for 17-Arm(20k)-PS. In the good solvent, *σ_f,e_* is lowered 13% for 17-Arm(2k)-PS, 34% for 17-Arm(6k)-PS, 31% for 17-Arm(10k)-PS, and 30% nm for 17-Arm(20k)-PS. The alterations of the star polymers due to the swelling are clearly discernible in the radial density profiles. These results collectively inform that high-molecular-weight 17-armed star polystyrene has a thicker fuzzy part in the Θ solvent, namely the unperturbed condition. However, such fuzzy part becomes thin in the good solvent; the thinning of fuzzy part due to the good solvent molecules is significantly pronounced by increasing the molecular weight of arm. Interestingly, a most amplified thinning of fuzzy part is achieved for 17-Arm(6k)-PS. These fuzzy part thinning behaviors may be attributed to the alteration of arm chain characteristics by the heavy association of good solvent molecules.

Fifth, more detailed structural information has been gained from the radial density profiles together with the *σ_f,e_* and *R_e_* data. From each radial density profile, the core radius *r_c,e_* is extracted directly, corresponding to the term [*R_e_* − 3*σ_f,e_*]; and the whole fuzzy part is estimated to have a thickness *t_f,e_*, which corresponds to 6*σ_f,e_*. In the Θ solvent, *r_c,e_* and *t_f,e_* are 1.46 and 4.14 nm for 17-Arm(2k)-PS, 1.89 and 5.94 nm for 17-Arm(6k)-PS, 2.46 and 8.64 nm for 17-Arm(10k)-PS, and 2.97 and 12.66 nm for 17-Arm(20k)-PS. In the good solvent, *r_c,e_* is increased 41% for 17-Arm(2k)-PS, 103% for 17-Arm(6k)-PS, 127% for 17-Arm(10k)-PS, and 171% for 17-Arm(20k)-PS; in contrast, *t_f,e_* is decreased for all star polymers discussed above. These results confirm again that in the good solvent, 17-armed star polymer is expanded in the core part but shrunk in the fuzzy shell part. However, the fuzzy shell part of the star polymer exhibits larger thickness than the core radius, regardless of the molecular weights and the solvent conditions.

Sixth, for each star polymer in a given solvent condition, the overall radius *R_t,e_* (i.e., total radius), including the full fuzzy region, is additionally determined from the radial density profile. Due to the presence of such fuzzy part, *R_t,e_* is larger by 3*σ_f,e_* against *R_e_*.

Seventh, the quantitative analysis has found the contribution of blobs (i.e., local density fluctuations inside the geometrical molecular volume of star polymer) to the measured X-ray scattering intensity profile, regardless of the molecular weight and the solvent conditions. Considering the molecular structure composed of the denser core and the less dense shell part in a density gradient, the blobs’ scatterings may occur mainly in the fuzzy shell part. The blob radius *ξ* (i.e., the average correlation length of local density fluctuations) is determined to range 0.40 to 2.60 nm in the Θ condition and 0.20 to 4.30 nm in the good solvent. In a given solvent, *ξ* exhibits some increment with increasing molecular weight. However, the *ξ* of 17-Arm(2k)-PS is significantly reduced in the good solvent. Interestingly, the *ξ* of 17-Arm(6k)-PS remains same in the good solvent. In contrast, those of 17-Arm(10k)-PS and 17-Arm(20k)-PS are increased in the good solvent respectively. Overall, the star polystyrenes could show no rationalized trend on the swelling dependency of *ξ* with good solvent.

Finally, each star polystyrene is found to have a size distribution (i.e., radius distribution). All star polystyrenes reveal single unimodal and narrow radius distributions, indicating that they were obtained in high purity through precise syntheses and subsequent purification processes. When they are compared, it is found that higher-molecular-weight star polystyrene shows relatively wider radius distribution. Furthermore, all star polystyrenes exhibit wider radius distributions in the good solvent, compared to those in the Θ condition.

**Figure 9:**
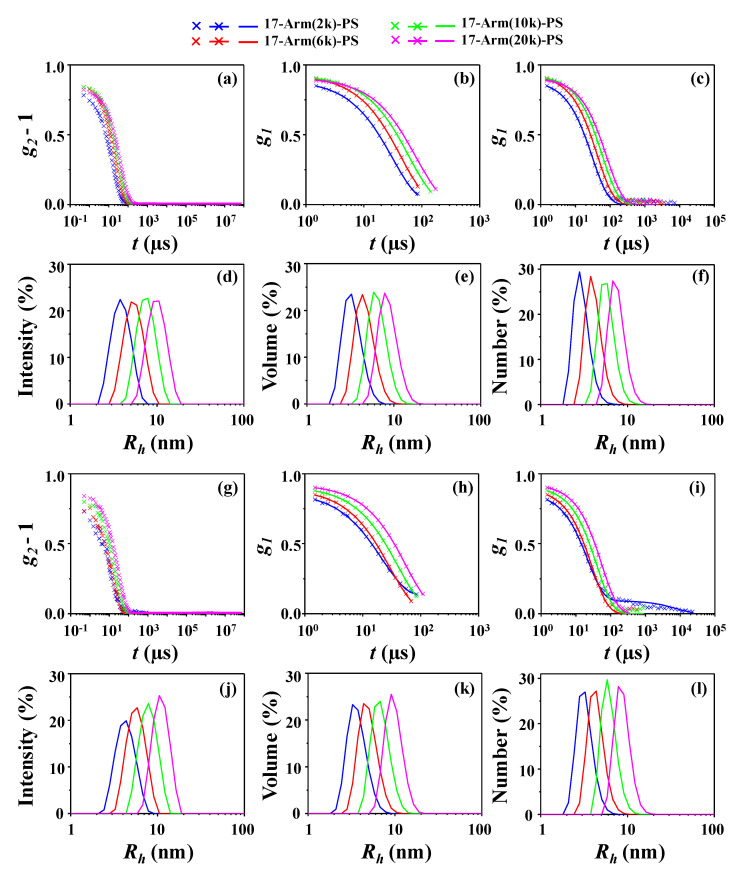
Dynamic light scattering (DLS) analyses of 17-armed star polystyrenes: (**a**) autocorrelation profiles measured in CHX at 35.0 °C (Θ condition); (**b**) analysis results of the data in (**a**), where the symbols are the measured data and the red solid lines were obtained by analysis using the cumulant method; (**c**) analysis results of the data in (**a**), where the symbols are the measured data and the red solid lines were obtained by analysis using the non-negatively constrained least square (NNLS) deconvolution algorithm; (**d**) intensity-weighted radius distributions obtained by the data analyses in (**c**); (**e**) volume-weighted radius distributions obtained from the radius distributions in (**d**); (**f**) number-weighted radius distributions obtained from the radius distributions in (**d**,**e**); (**g**) autocorrelation profiles measured in THF at 25.0 °C (good solvent); (**h**) analysis results of the data in (**g**), where the symbols are the measured data and the red solid lines were obtained by analysis using the cumulant method; (**i**) analysis results of the data in (**g**), where the symbols are the measured data and the red solid lines were obtained by analysis using the NNLS deconvolution algorithm; (**j**) intensity-weighted radius distributions obtained by the data analyses in (**i**); (**k**) volume-weighted radius distributions obtained from the radius distributions in (**j**); (**l**) number-weighted radius distributions obtained from the radius distributions in (**j,k**).

In addition, the star polystyrenes in both the Θ condition and the good solvent have been further characterized by DLS. The measured DLS data have been analyzed reasonably well by using the cumulant method [33,34] and non-negatively constrained least square (NNLS) deconvolution algorithm scheme [34,35], as shown in Figure 9. The obtained parameters are listed in Table 2.

The DLS analysis found that all star polymers of this study have single unimodal radius distributions, again confirming that they were prepared in high purity. The hydrodynamic radius *R_h,z_* (which were obtained by the analysis based on the cumulant method) ranges from 3.76 to 9.66 nm in the Θ condition and from 4.45 to 10.69 nm in the good solvent, depending on the molecular weights. Higher-molecular-weight star polystyrene reveals larger *R_h,z_* value. They exhibit larger *R_h,z_* values in the good solvent against those in the Θ condition. Overall, these characteristics and trends are in good agreement with those found by the X-ray scattering analysis. However, the radius distributions are much broader (namely, 362−850% broader) than those determined by the X-ray scattering analysis. The *R_h,z_* values are 20−60% larger than the *R_g_* values determined by the X-ray scattering analysis. The intensity-weighted averaged radius *R_h,intensity_* values (which were obtained by the analysis based on the NNLS deconvolution algorithm) are also found to be 24−57% larger than the *R_g_* values determined by the X-ray scattering analysis.

## 4. Conclusions

In this study, 17-armed star polystyrenes in various molecular weights were investigated in the Θ condition as well as in a solvent-swollen condition (good solvent) from the view of molecular structure and arm chain characteristics. Quantitative X-ray scattering analysis was successfully performed for the first time for the series of star polystyrenes in dilute solutions in a comprehensive manner, giving new insights into the 3D molecular structure details in the unperturbed condition and their alterations by swelling with good solvent molecules. The quantitative analysis results were further crosschecked in part by the DLS analysis as well as by the qualitative X-ray scattering analysis.

All 17-armed star polymers in the unperturbed state exhibit oblate fuzzy ellipsoids that are composed of a core (minor component: 19−26%) and a fuzzy shell (major component: 74−81%); the core fraction is the maximum (26%) for the lowest molecular weight star polymer, whereas the shell fraction is the maximum (81%) for the highest molecular weight star polymer. Nevertheless, the core becomes large and the fuzzy shell thickens, as the molecular weight increases. These structural characteristics are significantly altered in a good solvent. In particular, the core part is enlarged significantly, whereas the fuzzy shell is shrunk severely. These solvent-induced volume changes are obviously evidenced in the radial density profiles. The solvent-selective volume alterations of core and fuzzy shell parts in the opposite direction are quite unique. This exciting property together with relatively large fuzzy shell volume informs that 17-armed polystyrenes are very suitable for applications in various technological fields including smart deliveries of drugs, genes, bioimaging agents, and other desired chemicals.

## Figures and Tables

**Table 1:** Molecular characteristics and thermal properties of 17-armed star polystyrenes.

Star Polymer	Arm		Polymer	
	${\bar{M}}_{n}{\;}^{a}\;(g\;{\mathrm{mol}}^{-1})$	*Number ^b^*	${\bar{M}}_{w}{\;}^{c}\;(g\;{\mathrm{mol}}^{-1})$	*Đ ^d^*
17-Arm(2k)-PS	2300	17	45800	1.02
17-Arm(6k)-PS	6000	17	112000	1.02
17-Arm(10k)-PS	9500	17	167000	1.02
17-Arm(20k)-PS	20300	17	340000	1.02

*^a^* Number-averaged molecular weight measured by size exclusion chromatography (SEC) with polystyrene standards. *^b^* Number of polystyrene arms. *^c^* Weight-averaged molecular weight measured by static light scattering (SLS). *^d^* Polydispersity index measured by SEC analysis.

**Table 2:** Structural parameters of 17-armed star polystyrenes obtained by X-ray scattering and dynamic light scattering (DLS) analyses.

Structure Parameter	Star Polymers							
	17-Arm(2k)-PS		17-Arm(2k)-PS		17-Arm(2k)-PS		17-Arm(2k)-PS	
	CHX*^a^*	THF*^b^*	CHX	THF	CHX	THF	CHX	THF
Guinier analysis: [ln*I*(*q*) vs. *q*^2^ plot]								
*R_g,G_^c^* (nm)	2.67	2.80	3.75	4.12	5.20	6.05	7.18	8.52
*R_g,G_*(THF)/*R_g,G_*(Θ)		1.05		1.10		1.16		1.19
Kratky analysis-I: [*I*(*q*)*q*^2^vs. *qR_g,G_* plot]								
*q_max_R_g,G_^d^*	1.74	1.70	1.75	1.70	1.73	1.74	1.75	1.62
Kratky analysis-II [*I*(*q*)*q*^5/3^ vs. *qR_g,G_* plot]								
*q_max_R_g,G_^e^*	1.58	1.58	1.59	1.53	1.62	1.55	1.59	1.49
Kratky analysis-III [*I*(*q*)*q^n^* vs. *q* plot]								
*n ^f^*	4.64	4.80	4.39	4.43	3.87	3.92	3.82	3.92
Porod analysis: [*I*(*q*) vs. *q^−n^*plot]								
*n ^g^*	1.40	1.00	1.70	1.40	1.90	1.60	1.98	1.65
IFT analysis								
*R_g,IFT_^h^* (nm)	2.69	2.80	3.71	4.19	5.26	6.20	7.25	9.13
*R_max,IFT_^i^* (nm)	3.15	3.43	4.37	5.00	6.13	7.41	8.47	11.00
*D_max,IFT_^j^* (nm)	9.00	8.80	11.80	12.50	17.50	19.00	24.20	27.40
*R_max,IFT_/R_g,IFT_*	1.17	1.23	1.18	1.19	1.17	1.20	1.17	1.20
*D_max,IFT_/R_max,IFT_*	2.86	2.57	2.70	2.50	2.86	2.56	2.86	2.49
Model analysis								
*R_e_^k^*(nm)	3.53 (0.10) *^l^*	3.86 (0.14)	4.86 (0.18)	5.79 (0.25)	6.78 (0.29)	8.55 (0.34)	9.30 (0.41)	12.50 (0.50)
*σ_f,e_^m^*(nm)	0.69	0.60	0.99	0.65	1.44	0.99	2.11	1.48
*ξ ^n^* (nm)	0.40	0.20	0.80	0.80	1.60	1.90	2.60	4.30
*R_av_^o^*(nm)	2.95	3.22	4.05	4.84	5.56	7.13	7.63	10.58
*ε ^p^*	0.51	0.50	0.50	0.51	0.46	0.50	0.46	0.54
*R_t,e_^q^*(nm)	5.60	5.66	7.83	7.74	11.10	11.52	15.63	16.94
*r_c,e_^r^*(nm)	1.46	2.06	1.89	3.84	2.46	5.58	2.97	8.06
*t_f,e_^s^*(nm)	4.14	3.60	5.94	3.90	8.64	5.94	12.66	8.88
*R_av,t_^t^*(nm)	4.69	4.72	6.53	6.48	9.10	9.60	12.82	14.34
*r_c,e_*/*R_t,e_*	0.26	0.36	0.24	0.50	0.22	0.48	0.19	0.48
*t_f,e_*/*R_t,e_*	0.74	0.64	0.76	0.50	0.78	0.52	0.81	0.52
*R_g_^u^* (nm)	2.65	2.78	3.67	4.10	5.14	6.06	7.14	8.92
*R_max_^v^*(nm)	3.13	3.34	4.30	4.95	5.96	7.30	8.24	10.90
*D_max_^w^* (nm)	9.27	7.91	12.64	12.44	17.24	21.77	23.65	30.90
*R_g_*/*R_g,G_*	0.99	0.99	0.98	1.00	0.99	1.00	0.99	1.05
*R_g_*/*R_g,IFT_*	0.99	0.99	0.99	0.98	0.98	0.98	0.98	0.98
*R_max_*/*R_max,IFT_*	0.99	0.97	0.98	0.99	0.97	0.99	0.97	0.99
*D_max_*/*D_max,IFT_*	1.03	0.90	1.07	1.00	0.99	1.15	0.98	1.13
DLS Analysis								
*R_h,z_^x^* (nm)	3.76	4.45	5.23	5.47	7.31	7.60	9.66	10.69
*PDI_DLS_^y^*	0.038	0.273	0.024	0.026	0.013	0.034	0.024	0.009
*R_h,intensity_^z^* (nm)	3.96 (0.95)	4.37 (1.06)	5.52 (1.33)	5.79 (1.34)	7.63 (1.76)	8.00 (1.78)	10.10 (2.38)	11.10 (2.31)
*R_h,voulme_^aa^* (nm)	3.39 (0.85)	3.73 (0.95)	4.71 (1.20)	4.99 (1.24)	6.60 (1.63)	6.98 (1.68)	8.70 (2.19)	9.81 (2.26)
*R_h,number_^ab^* (nm)	3.00 (0.65)	3.28 (0.74)	4.13 (0.93)	4.40 (0.98)	5.83 (1.29)	6.23 (1.32)	7.66 (1.71)	8.81 (1.86)
*R_h,z_/R_g_*	1.42	1.60	1.43	1.33	1.42	1.25	1.35	1.20
*R_h,z_/R_e_*	1.06	1.15	1.08	0.94	1.08	0.89	1.04	0.86
*R_h,z_/R_t,e_*	0.67	0.79	0.67	0.71	0.66	0.66	0.62	0.63
*R_h,intensity_/R_g_*	1.49	1.57	1.50	1.41	1.48	1.32	1.41	1.24
*R_h,intensity_/R_e_*	1.12	1.13	1.14	1.00	1.13	0.94	1.09	0.89
*R_g,intensity_/R_t,e_*	0.71	0.77	0.70	0.75	0.69	0.69	0.65	0.66

*^a^* Measured in cyclohexane (CHX) at 35.0 °C (Θ condition). *^b^* Measured in tetrahydrofuran (THF) at 25.0 °C (good solvent condition). *^c^* Radius of gyration determined from Guinier analysis. *^d^* Determined from Kratky analysis. *^e^* Determined from a modified Kratky analysis. *^f^* Exponent determined from another modified Kratky analysis. *^g^* Exponent determined from Porod analysis. *^h^* Radius of gyration determined from indirect Fourier transformation (IFT) analysis. *^i^* Radius determined from the peak maximum of the *p*(*r*) function in IFT analysis. *^j^* Maximum dimension determined from the *p*(*r*) function in IFT analysis. *^k^* Mean radius of isotropic ellipsoid along the equatorial direction. *^l^* Standard deviation. *^m^* Characteristic parameter for the thickness of fuzzy shell in fuzzy ellipsoid along the equatorial direction. *^n^* Average correlation length of density fluctuation (i.e., blob radius) in fuzzy ellipsoid (i.e., star polymer molecule). *^o^* Average radius of isotropic ellipsoid. *^p^* Ellipsoidicity ratio (polar radius/equatorial radius). *^q^* Mean radius (i.e., total radius) of fuzzy ellipsoid along the equatorial direction. *^r^* Mean core radius of fuzzy ellipsoid along the equatorial direction. *^s^* Mean thickness of fuzzy shell in fuzzy ellipsoid along the equatorial direction. *^t^* Averaged total radius of fuzzy ellipsoid. *^u^* Radius of gyration determined from quantitative model analysis. *^v^* Radius determined from the peak maximum of the *p*(*r*) function obtained by quantitative model analysis. *^w^* Maximum dimension determined from the *p*(*r*) function obtained by quantitative model analysis. *^x^* z-Averaged hydrodynamic radius. *^y^* Polydispersity index of hydrodynamic radius. *^z^* Intensity-weighted mean radius. *^aa^* Volume-weighted mean radius. *^ab^* Number-weighted mean radius.

## Supplementary Materials

The following are available online at https://www.mdpi.com/2073-4360/12/9/1894/s1, Figure S1. Pair distance distribution functions *p*(*r*) obtained from numerical Fourier transformation of the extrapolated scattering intensity profiles obtained by the model analysis: (a, b) CHX at 35.0 °C (Θ condition); (c, d) THF at 25.0 °C (good solvent).
