# Supplementary material for: Seventeen-Armed Star Polystyrenes in Various Molecular Weights: Structural Details and Chain Characteristics
Source: Polymers (Basel). 2020 Aug 23;12(9):1894. doi: 10.3390/polym12091894 (PMC7563263; doi:10.3390/polym12091894)
Supplement: Supplementary file 1 [file polymers-12-01894-s001.pdf]

Supplementary Information

Seventeen-Armed Star Polystyrenes in Various Molecular Weights: Structural Details and Chain Characteristics

Jia Chyi Wong,[1,2] Li Xiang,[2] Kuan Hoon Ngoi,[1,2] Chin Hua Chia,[*,1] Kyeong Sik Jin,[*,3] Akira Hirao,[*,4,5] and Moonhor Ree[*,2]

[1] Materials Science Program, Department of Applied Physics, Faculty of Science and Technology, Universiti Kebangsaan Malaysia, 43600 Bangi, Selangor, Malaysia; wongjiachyi@gmail.com (J.C.W.); ngoikuanhoon@gmail.com (K.H.N.); chia@ukm.edu.my (C.H.C.)

[2] Department of Chemistry and Pohang Accelerator Laboratory, Pohang University of Science and Technology, Pohang 37673, Republic of Korea; lea1990@postech.ac.kr (L.X.); ree@postech.edu (M.R.)

[3] Pohang Accelerator Laboratory, Pohang University of Science & Technology, Pohang 37673, Republic of Korea; jinks@postech.ac.kr (K.S.J.)

[4] Department of Chemical Science and Engineering, Graduate School of Materials and Chemical Engineering, Tokyo Institute of Technology, 2-12-1-S1-13, Ohokayama, Meguro-ku, Tokyo 152-8550, Japan; ahirao@email.plala.or.jp (A.H.)

[5] Department of Chemical Engineering, National Taiwan University, No. 1, Sec. 4, Roosevelt Road, Taiwan; ahirao@email.plala.or.jp (A.H.)

X-ray Scattering Data Analysis

The scattering intensity profiles $I(q)$ of the star polystyrenes in dilute solution have been analyzed by several analysis schemes below.

The Guinier law is expressed in the equations below [1,2]:

$$I(q) = I_0 \exp\left(-\frac{q^2 {R_{g,G}}^2}{3}\right) \quad (1)$$

$$\ln[I(q)] = \ln[I_0] - \left(\frac{{R_{g,G}}^2}{3}\right) q^2 \quad (2)$$

where q is the scattering vector, $I(q)$ is the scattering intensity at certain q, I_0 is the scattering intensity at $q = 0$. By using the Guinier plot which is the linear plot of ln $[I(q)]$ versus q^2 from the SAXS data, the slope in the low q region, $-\frac{{R_{g,G}}^2}{3}$ can be used to calculate the radius of gyration, $R_{g,G}$ of the sample. The obtained radius of gyration indicates the effective size of the scattering object regardless of the form. However, it is more accurate for nearly isodiametric

sample and calculated from slope at very low q region. In this study, all the slopes were obtained at maximum $qR_g \leq 1.3$.

The Porod plot is a linear plot of log $I(q)$ versus log q [2-4]:

$$I(q) = \frac{A}{q^n} + B \tag{3}$$

$$\log\,[I(q) - B] = \log A - n \log q \tag{4}$$

where A is the arbitrary constant, B is the constant scattering background and n is the Porod exponent. The Porod exponent can be obtained from the slope of the plot and it gives clues to the nature of local structure of scatterer. Porod exponent $n = 1$ indicates the scattering from stiff rods, $n = 5/3$ indicates swollen chains in good solvent, $n = 2$ indicates Gaussian chains in theta solvent or two-dimensional structure, $n = 3$ indicates collapsed chains in bad solvent or particles with rough surface while $n = 4$ indicates particles with smooth surface.

Besides, the Porod exponent can be clearly visualized in the Kratky plot or modified Kratky plot, log $[I(q)\, q^{\mathrm{n}}]$ versus qR_g [3]. A horizontal asymptote would be shown in the high q region. In this study, modified Kratky plots with $n = 5/3$ and $n = 2$ were applied for scattering data. $n = 5/3$ is an indication for the characteristic of swollen chain; $n = 2$ is an indication for Gaussian chain.

The indirect Fourier transformation (IFT) method can analyze the scattering intensity $I(q)$ from particles in a solution [3,5-11]. With the priori information of maximum dimension D_{max}, the radius of gyration $R_{g,IFT}$ and radius at peak maximum R_{max} can be obtained from the pair distance distribution function $p(r)$ by assuming the $p(r)$ as the series below:

$$p(r) = \textstyle\sum_{i=1}^{N} c_i\, \varphi_i(r) \qquad \text{for } 0 \leq r \leq D_{max} \tag{5}$$

where N is the number of spline functions, c_i are the unknown expansion coefficients and $\varphi_\mathrm{i}(r)$ are the cubic B splines. Due to the linearity of Fourier transformation,

$$I(q) = \textstyle\sum_{i=1}^{N} c_i\, \psi_\mathrm{i}(q) \tag{6}$$

where $I(q)$ is the Fourier transform of $p(r)$ and is represented by the series of Fourier transformed B splines $\psi_i(q)$. Under the constraint of N_c,

$$N_c = \sum_{i=1}^{N-1} (c_{i+1} - c_i)^2 \tag{7}$$

together with the Lagrange multiplier, λ under least square condition,

$$(L+\lambda \cdot N_c) = \text{Min} \tag{8}$$

c_i is then calculated by weighted least-squares operation:

$$L = \frac{1}{M}\sum_{k=1}^{M} \frac{[I_{exp}(q_k) - I(q_k)]^2}{\sigma^2(q_k)} = \frac{1}{M}\sum_{k=1}^{M} \frac{[I_{exp}(q_k) - \sum_{i=1}^{N} c_i\, \psi_i(q_k)\,]^2}{\sigma^2(q_k)} \tag{9}$$

where L is the mean deviation between experimental scattering intensity $I_{exp}(q_k)$ and the approximated solution function $I(q_k)$, M is the number of experimental points and $\sigma(q_k)$ is the standard deviation of the input data $I_{exp}(q_k)$.

Thus, the $p(r)$ profile is then obtained with the optimized c_i indirectly and subsequently, the radius of gyration, $R_{g,\,IFT}$ of the particle can be estimated by using the following relation:

$$R_{g,\,IFT}^{\;2} = \frac{\int p(r)\, r^2\, \mathrm{d}r}{2\int p(r)\, \mathrm{d}r}\ . \tag{10}$$

In fact, the scattering intensity of particles in a solution can be expressed by the equation below [5-7,12,13]:

$$I(q) = n_p\, P(q)\, S(q) \tag{11}$$

where $n_p = N_p/V$ is the number density of particles in the sample where N_p is the number of particles and V is the solution sample volume exposed to the incident beam of X-ray, $P(q)$ is the form factor of the particle, and $S(q)$ is the structure factor for the particles. The intensity is measured as a function of scattering vector $q = (4\pi \sin \theta)/ \lambda$, where 2θ is the scattering angle and λ is the wavelength of the X-ray. In this study, diluted star polystyrene polymer solutions were used in X-ray scattering measurements, n_p is assumed as arbitrary constant N and $S(q)$

term can be approximated to be unity over the entire q range. Therefore, equation (5) can be written as [5-7,14]:

$$I(q) = NP(q)\,. \tag{12}$$

The fuzzy ellipsoid model has been developed to account a star polystyrene with mean equatorial radius, R_e that is not in perfect sphere and contains region of fuzzy interface (approximately $6\sigma_{f,e}$) between star polystyrene and solvent with gradual decrease of radial scattering length density distribution [5-8,15]. The geometry of the ellipsoidal particle can be defined by the ellipsoidicity ratio (ε) between respective polar radius (r_p) and equatorial radius (r_e) as below:

$$\varepsilon = \frac{r_p}{r_e} \tag{13}$$

where sphere is obtained when $r_e = r_p$ (i.e., $\varepsilon = 1$), prolate ellipsoid is obtained when $r_e < r_p$ (i.e., $\varepsilon > 1$) and oblate ellipsoid is obtained when $r_e > r_p$ (i.e., $\varepsilon < 1$).

This model considers the convolution of scattering amplitudes (Φ) from ellipsoid of revolution with Gaussian smoothing function, which can be expressed by [15]:

$$A\left(q, r'(\alpha, r_e)\right) = 3\left[\frac{\sin(qr') - qr'\cos(qr')}{(qr')^3}\right] \cdot \exp\left(-\left(\frac{q\sigma_{f,e} r_e}{\sqrt{2}\,R_e}\right)^2\right)\,. \tag{14}$$

where r' is the effective radius of ellipsoid defined by the r_e and r_p in radians of $0 \leq \alpha \leq \pi/2$ as below:

$$r'(\alpha, r_e) = r_e \sqrt{\sin^2\alpha + \varepsilon^2 \cos^2\alpha}\,. \tag{15}$$

Subsequently, the form factor is expressed as below:

$$P\left(q, r'(\alpha, R_e)\right) = \left[A\left(q, r'(\alpha, R_e)\right)\right]^2. \tag{16}$$

To consider polydisperse star polystyrenes in the solution, the Gaussian distribution function is assumed [7]:

$$n(r_e) = \frac{1}{\sigma_R\sqrt{2\pi}}\exp\left(-\frac{(r_e - R_e)^2}{2\sigma_R{}^2}\right) \tag{17}$$

where R_e is the mean equatorial radius in the distribution and σ_R is the standard deviation for R_e. Hence, the number-averaged intensity of ellipsoidal star polystyrenes with random orientation in dilute solution is expressed as below:

$$I_e(q, r_e) = N\int_0^{\frac{\pi}{2}}\int_0^{\infty} n(r_e)\,(\Delta\rho V(r_e))^2\, P\left(q, r'(\alpha, r_e)\right) \mathrm{d}r_e \sin\alpha\, \mathrm{d}\alpha\,. \tag{18}$$

where $\Delta\rho = (\rho - \rho_{solv})$, ρ is the average radial scattering length density of star polystyrene, ρ_{solv} is the average radial scattering length density of the solvent and $V(r_e)$ is the volume of ellipsoid which is equal to $4\pi\varepsilon r_e^3/3$.

To consider the intensity contribution arising from density fluctuations on length scales smaller than the blob radius ξ within particle, an additional intensity $I_{blob}(q)$ in its integral form can be expressed as below [8,16]:

$$I_{blob}(q) = a_b' 4\pi \int_0^{\bar{\xi}} r^2\gamma(r)\frac{\sin(qr)}{qr}\mathrm{d}r \tag{19}$$

while the analytical form can be expressed as below:

$$I_{blob}(q) = \left(\frac{a^b}{\mu q_b^*}\right)\frac{\sin\left(\mu\tan^{-1}(q_b^*)\right)}{\left(1+q_b^{*2}\right)^{\mu/2}} \tag{20}$$

where $a_b = a_b' 4\pi\,\mu\,\bar{\xi}^{\mu+1}\Gamma(\mu)$ so that $\frac{P_a(q\to 0)}{a_b}=1$, a_b' is the relative amplitude of the blob scattering contribution, $\Gamma(\mu)$ is Gamma function; $\mu=\frac{1}{v} - 1$ where v denotes the Flory-Huggins parameter which equal to 3/5 for good solvent condition, 1/2 for theta solvent and 2/3 for stretching of the polymer segment; $q_b^* = q^*\bar{\xi}$ and $q^* = \frac{q}{\left(\mathrm{erf}(\frac{qR_g}{\sqrt{6}})\right)^3}$ where q^* is the reduced q to ensure that the

contribution of the ellipsoid form factor dominates at the low q region in the scattering intensity profile while contribution of the blob scattering dominates only at the high q region of the scattering intensity profile.

Therefore, the total scattering intensity is expressed as below [7,8,16]:

$$I(q) = I_e(q, r_e) + I_{blob}(q) . \tag{21}$$

Moreover, the radial scattering length density profile $\Delta\rho(r)$ along the mean equatorial radius R_e of single particle can be further obtained by numerical Fourier transformation of the respective scattering amplitudes [8,15]:

$$\Delta\rho(r) = \frac{1}{2\pi^2}\int A\left(q, r'(\alpha, R_e)\right)\frac{\sin(qr)}{qr}\mathrm{d}q \tag{22}$$

where $r'(\alpha, R_e) = R_e$. In this study, the model generated scattering amplitude is extrapolated to the range of $5 < q \leq 35$ nm^{-1} from the measurable range of $0.07 \leq q \leq 5$ nm^{-1} in order to avoid termination ripples due to the insufficient experimental range of q. The extrapolated range of q lower than 0.07 nm^{-1} contributes insignificantly to the transformed $\Delta\rho(r)$.

In addition, the pair distance distribution function $p(r)$ can also be obtained from numerical Fourier transformation of the extrapolated intensity profile $I(q)$ determined by structural model analysis (Figure S1):

$$p(r) = \frac{1}{2\pi}\int I(q)\, qr\sin(qr)\,\mathrm{d}q . \tag{23}$$

In this study, the model generated $I(q)$ is extrapolated to the range of $0.001 \leq q < 0.07$ nm^{-1} and $5 < q \leq 300$ nm^{-1} from the measurable range of $0.07 \leq q \leq 5$ nm^{-1} to avoid termination ripples due to the insufficient experimental range of q.

REFERENCES

1. Guinier, A.; Fournet, G.; Yudowitch, K. L. *Small-angle scattering of X-rays*. 1955, New York: John Wiley & Sons, Inc.

2. Hammouda, B. *A*nalysis of the Beaucage model. *J. Appl. Crystallogr.* **2010**, *43*, 1474-1478.
3. Glatter, O.; Kratky, O. *Small angle X-ray scattering*. 1982: Academic press.
4. Hammouda, B. *Chapter 22: Standard Plots.* The Sans Toolbox, 2010.
5. Ree, B. J. et al. A Comparative Study of Dynamic Light and X-ray Scatterings on Micelles of Topological Polymer Amphiphiles. *Polymers* **2018**, *10*, 1347.
6. Ree, B. J.; Satoh, T.; Yamamoto, t. Micelle Structure Details and Stabilities of Cyclic Block Copolymer Amphiphile and Its Linear Analogues. *Polymers* **2019**, *11*, 163.
7. Ree, B. J., et al. Well-defined and stable nanomicelles self-assembled from brush cyclic and tadpole copolymer amphiphiles: a versatile smart carrier platform. *NPG Asia Materials* **2017**, *9*, e453.
8. Rathgeber, S.; Monkenbusch, M.; Kreitschmann, M.; Urban, V.; Brulet, A. Dynamics of star-burst dendrimers in solution in relation to their structural properties. *J. Chem. Phys.* **2002**. *117*, 4047-4062.
9. Glatter, O. A new method for the evaluation of small angle scattering data. *J. Appl. Crystallogr.* **1977**, *10*, 415-421.
10. Mittelbach, R.; Glatter, O. Direct Structure Analysis of Small-Angle Scattering Data from Polydisperse Colloidal Particles. J. Appl.Crystallogr. **1998**, *31*, 600-608.
11. Müller, K.; Glatter, O. *Practical aspects to the use of indirect fourier transformation methods. Makromol. Chem.* **1982**, *183*, 465-479.
12. Li, T.; Senesi, A. J.; Lee, b. Small Angle X-ray Scattering for Nanoparticle Research. *Chem. Rev*. **2016**, *116*, 11128-11180.
13. Pedersen, J. S. *A*nalysis of small angle scattering data from colloids and polymer solutions: modeling and least squares fitting. *Adv. Colloid Interface Sci.* **1997**, *70*, 171-210.
14. Kotlarchyk, M.; Chen, S. H. Analysis of small angle neutron scattering spectra from polydisperse interacting colloids. *J. Chem. Phys.* **1983**, *79*, 2461-2469.
15. Stieger, M.; Richtering, W.; Pedersen, J. S.; Lindner, P. Small-angle neutron scattering study of structural changes in temperature sensitive microgel colloids. *J. Chem .Phys.* **2004**, *120*, 6197-206.
16. Dozier, W. D.; Huang, J. S.; Fetters, L. J. Colloidal nature of star polymer dilute and semidilute solutions. *Macromolecules* **1991**, *24*, 2810-2814

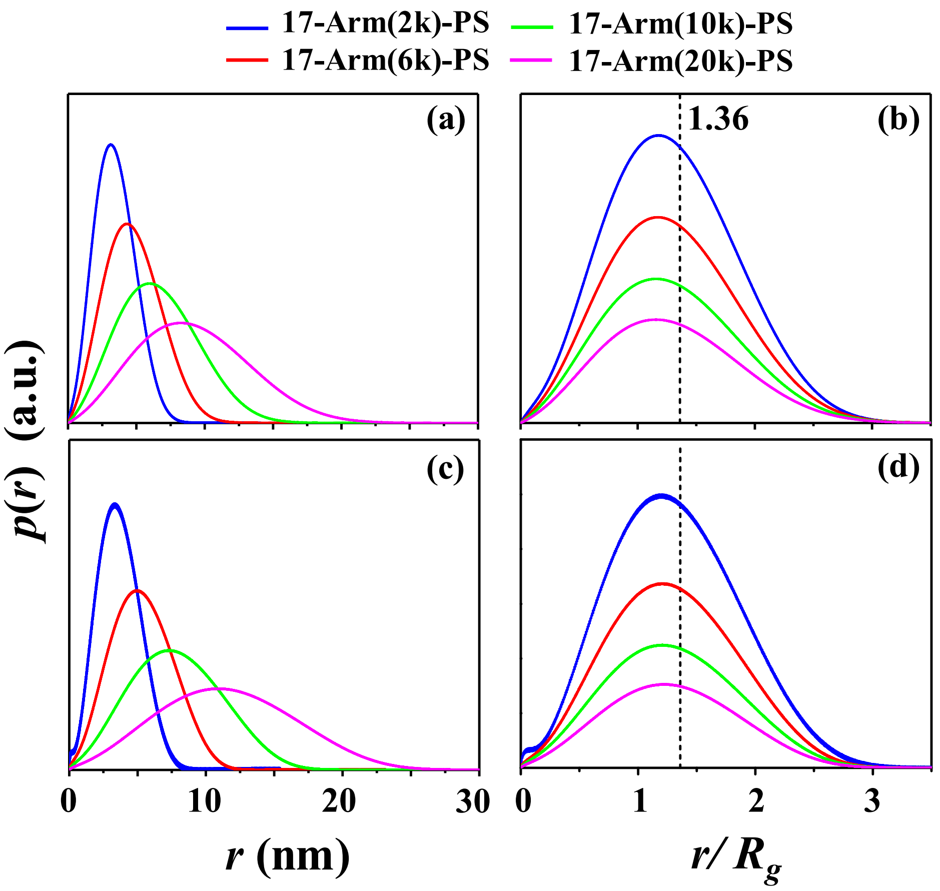

Figure S1. Pair distance distribution functions $p(r)$ obtained from numerical Fourier transformation of the extrapolated scattering intensity profiles obtained by the model analysis: (a, b) CHX at 35.0 °C (Θ condition); (c, d) THF at 25.0 °C (good solvent).
